# Different responses of soil respiration to environmental factors across forest stages in a Southeast Asian forest

**DOI:** 10.1002/ece3.8248

**Published:** 2021-10-19

**Authors:** Chadtip Rodtassana, Weerapong Unawong, Siriphong Yaemphum, Wirong Chanthorn, Sakonvan Chawchai, Anuttara Nathalang, Warren Y. Brockelman, Pantana Tor‐ngern

**Affiliations:** ^1^ Department of Botany Faculty of Science Chulalongkorn University Bangkok Thailand; ^2^ Center of Excellence on Hazardous Substance Management Chulalongkorn University Bangkok Thailand; ^3^ Graduate School Chulalongkorn University Bangkok Thailand; ^4^ Department of Environmental Technology and Management Faculty of Environment Kasetsart University Bangkok Thailand; ^5^ Department of Ecological Modelling Helmholtz Centre for Environmental Research UFZ Leipzig Germany; ^6^ Department of Geology Faculty of Science Chulalongkorn University Bangkok Thailand; ^7^ National Biobank of Thailand National Science and Technology Development Agency Pathum Thani Thailand; ^8^ Institute of Molecular Biosciences Mahidol University Nakhon Pathom Thailand; ^9^ Department of Environmental Science Faculty of Science Chulalongkorn University Bangkok Thailand; ^10^ Water Science and Technology for Sustainable Environment Research Group Chulalongkorn University Bangkok Thailand; ^11^ Environment, Health and Social Data Analytics Research Group Chulalongkorn University Bangkok Thailand

**Keywords:** forest succession, soil moisture, soil organic matter, soil respiration, soil temperature, tropical forests

## Abstract

Soil respiration (SR) in forests contributes significant carbon dioxide emissions from terrestrial ecosystems and is highly sensitive to environmental changes, including soil temperature, soil moisture, microbial community, surface litter, and vegetation type. Indeed, a small change in SR may have large impacts on the global carbon balance, further influencing feedbacks to climate change. Thus, detailed characterization of SR responses to changes in environmental conditions is needed to accurately estimate carbon dioxide emissions from forest ecosystems. However, data for such analyses are still limited, especially in tropical forests of Southeast Asia where various stages of forest succession exist due to previous land‐use changes. In this study, we measured SR and some environmental factors including soil temperature (ST), soil moisture (SM), and organic matter content (OM) in three successional tropical forests in both wet and dry periods. We also analyzed the relationships between SR and these environmental variables. Results showed that SR was higher in the wet period and in older forests. Although no response of SR to ST was found in younger forest stages, SR of the old‐growth forest significantly responded to ST, plausibly due to the nonuniform forest structure, including gaps, that resulted in a wide range of ST. Across forest stages, SM was the limiting factor for SR in the wet period, whereas SR significantly varied with OM in the dry period. Overall, our results indicated that the responses of SR to environmental factors varied temporally and across forest succession. Nevertheless, these findings are still preliminary and call for detailed investigations on SR and its variations with environmental factors in Southeast Asian tropical forests where patches of successional stages dominate.

## Graphical abstract

We measured and analyzed soil respiration (SR) with environmental factors in three successional forests in Thailand. Results showed that SR was generally higher in the wet season and in older forests. Across all forest stages, soil moisture was the main factor contributing to variation in SR in the wet season while organic matter content played significant role in the dry season.

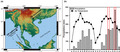

## 1 INTRODUCTION

The role of climate change in the functioning of forests has been increasingly recognized by the global community. Forests cover about 30% of the global land surface and store ~45% of terrestrial carbon (Bonan, 2008). Global forests sequester and store carbon in above‐ and below‐ground parts (Bunker et al., 2005; Giardina et al., 2004), and they release carbon dioxide (CO_2_) back into the atmosphere through respiration by plants and soil. Soil respiration (SR) is an important component of the global carbon cycle, contributing 78–95 Pg of carbon back into the atmosphere annually (Bond‐Lamberty & Thomson, 2010; Hashimoto et al., 2015). Specifically, SR in forests represents 40–90% of total CO_2_ emissions from terrestrial ecosystems (Granier et al., 2000; Schlesinger & Andrews, 2000).

Soil respiration is highly sensitive to environmental change because it is influenced by many factors including soil temperature, soil moisture, the microbial community, surface litter, and vegetation type (Davidson et al., 2006; Fekete et al., 2014; Grace, 2004; Jenkinson et al., 1991; Yan et al., 2006). In fact, even small changes in SR can incur profound impacts on the global carbon balance, further affecting feedbacks to climate change (Davidson et al., 2006). Despite several studies on SR and its drivers in forests in boreal and temperate regions, such investigations remain elusive in tropical systems, especially in Southeast Asia. Deforestation and land‐use change are particularly pervasive across Southeast Asia (FAO & UNEP, 2020; Zeng et al., 2018), where large‐scale agricultural production and commercial tree plantations are the main drivers of forest loss (Curtis et al., 2018). However, due to unsustainable practices, such large‐scale operations have often been abandoned, leading to the regeneration of secondary forests naturally or artificially. Consequently, forests in Southeast Asia are mostly characterized by patches of primary, old‐growth forest and forests at different stages of secondary succession. Such variations in forests may exert different impacts on SR through modifications of environmental factors associated with successional gradients.

Forest succession often modifies microclimatic conditions and biogeochemical cycles (De Kovel et al., 2000; Lebrija‐Trejos et al., 2011; Li et al., 2013) and varies with species composition and abundance (Sheil, 2001). Therefore, the driving factors for SR are affected by the forest succession (Raich & Tufekcioglu, 2000). For instance, soil organic carbon, total nitrogen, and microbial biomass increase rapidly with secondary forest succession (Jia et al., 2005). The rate of surface litter decomposition has been found to be higher in older successional stages of tropical dry secondary forests (Tolosa et al., 2003). Although several studies have investigated SR and its driving factors in association with forest succession (Gao et al., 2020; Han et al., 2015; Huang et al., 2016; Luo et al., 2012; Wang et al., 2017; Yan et al., 2006, 2009), none of these studies were conducted in tropical forests of Southeast Asia.

To help fill this knowledge gap, we measured SR of three successional forest plots in a seasonal evergreen forest in Thailand. We performed the measurements in the wet (June and September 2020) and the dry (February and March 2021) periods within plots of different stages of succession: young forest (YF, ~5 years), intermediate forest (IF, ~45 years), and old‐growth forest (OF, >200 years). The main research questions included the following:(1) Does SR differ across successional forests and among periods of data collection? and (2) Does SR respond to environmental factors including soil organic matter (OM), soil temperature (ST), and soil moisture (SM) and whether these relationships (if any) differ across forest stages? Note that we did not intend to estimate total carbon dioxide efflux from these forests, but rather aimed to investigate the dynamical changes of SR in response to various environmental factors across forest succession.

## 2 MATERIALS AND METHODS

### 2.1 Site description

**FIGURE 1:**
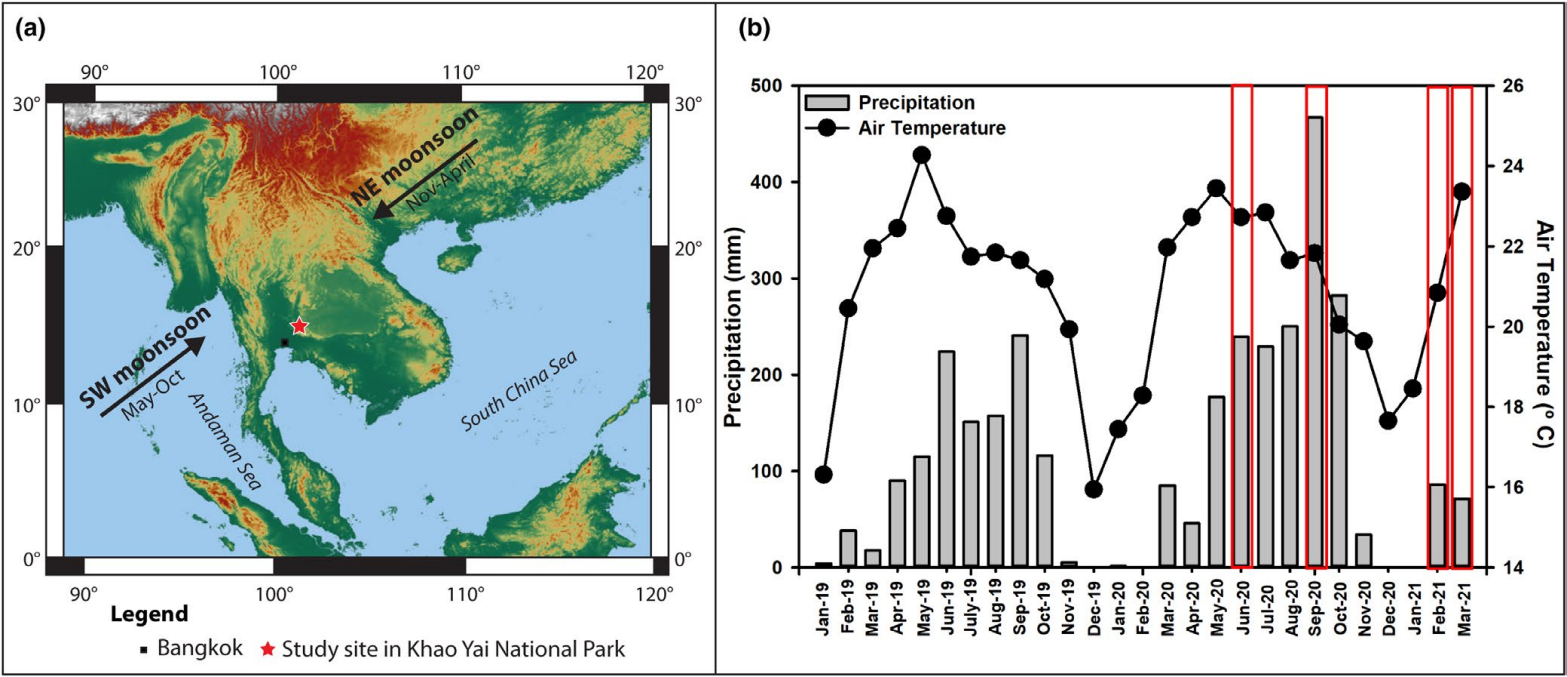
(a) Location of Khao Yai National Park in Thailand where the study was performed. (b) Monthly total precipitation (mm; bars) and average air temperature (°C; circles) profiles in Khao Yai National Park. Data from January 2019 to December 2020 were obtained from a local station near the old‐growth forest (OF), whereas those from January to March 2021 were from the weather station near the young forest (YF). Red boxes indicate the months in which our measurements were made

The study was conducted in seasonal evergreen forest at 700–800 m asl in Khao Yai National Park (KYNP), Nakhon Ratchasima Province, Thailand (14°26ʹ31ʺN, 101°22ʹ55ʺE; Figure 1a). According to data spanning 1994–2018, mean annual temperature and precipitation at the site are 22.4 °C and 2,100 mm, respectively (Department of National Parks, Wildlife and Plant Conservation; 25‐year means). The wet season lasts from May to October and the dry season from about late October to April, when monthly precipitation is less than 100 mm (Brockelman et al., 2017). During the study, precipitation peaked in September, which accounted for 21% (2019) and 26% (2020) of total precipitation in the wet season (data from a rain logger near the study site). Monthly precipitation was 239.2 mm (June 2020), 466.9 mm (September 2020), 33.6 mm (February 2021), and 1.4 mm (March 2021), as shown in Figure 1b. Using the same criteria as in Brockelman et al. (2017), we identified two data collection periods: the wet period in June through September 2020, when monthly precipitation exceeded 100 mm, and the dry period in February and March 2021.

**FIGURE A1:**
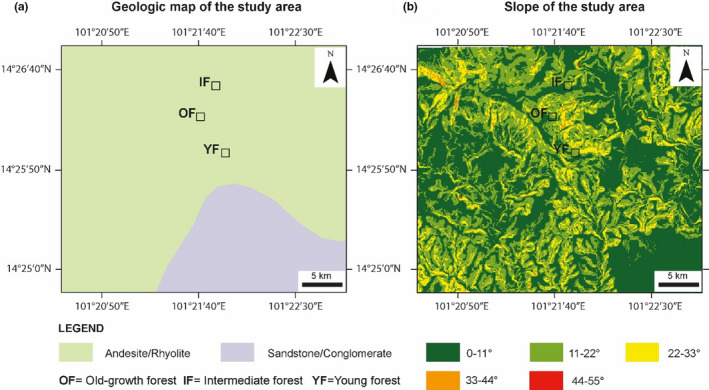
(a) Geologic map and (b) Slope of the study area and location of the study sites

KYNP contains mostly old‐growth (primary) forest with scattered patches of secondary forest at various stages, which have regenerated from old fields within the past 50 years (Jha et al., 2020). For this study, we selected three plots representing different stages. The first plot was within the 30‐ha Mo Singto forest dynamic plot (Brockelman et al., 2017), a ForestGEO plot in the global network of the Center for Tropical Forest Science (CTFS), Smithsonian Tropical Research Institute (Davies et al., 2021). CTFS plots are established using a uniform methodology (Condit, 1998) in which every woody stem ≥1 cm DBH is identified, mapped, and measured every 5 years. This plot represented an old‐growth stage (hereafter OF), with the age of at least ca. 200 years. The OF’s mean canopy height was 30 m with some emergent trees higher than 50 m, a leaf area index (LAI) of 5, and stem density of 1,112 trees ha^−1^ (Brockelman et al., 2017; Chanthorn et al., 2016). Adjacent to the northern edge of this plot, a 1‐ha plot in a secondary forest was established in 2003, using the same CTFS methods. This plot (hereafter IF) was at an intermediate successional stage about 45 years of age, classified as the stem exclusion stage. The forest canopy of IF was more uniform and denser than that of and had a mean canopy height of 25 m, an LAI of 6, and stem density of 2,052 trees ha^−1^ (Chanthorn et al., 2016). About 3 km away from the OF plot, we established a 2‐ha plot in a 5‐year‐old, initial stage forest (hereafter YF). Its mean canopy height was 15 m, and stem density was 1,226 trees ha^−1^. Despite the lack of LAI data, the YF canopy was distinctly sparse compared with the other stages based on visual observation. The soil type of these forests was gray, brown ultisol, but the soils under the IF and YF were degraded by shifting agriculture and burning prior to regeneration (Chanthorn et al., 2016, 2017). Based on the preliminary measurement at the sites, bulk density of the soil in IF (averaged 0.93 g cm^−3^) was lower than that in OF and YF (1.26 and 1.24 g cm^−3^, respectively). The soil texture at the study plots, measured at 10 cm depth, was classified as sandy clay‐loam and clay loam with the highest sand contents in YF plots measured in September 2020 and February 2021 as 64.4 ± 3.06% and 56.4 ± 5.03%, respectively (Appendix A, Table A1). All study sites (OF, IF, and YF) are similar with respect to geology and slope (Appendix A, Figure A1).

### 2.2 Measurements of the study variables

**FIGURE A2:**
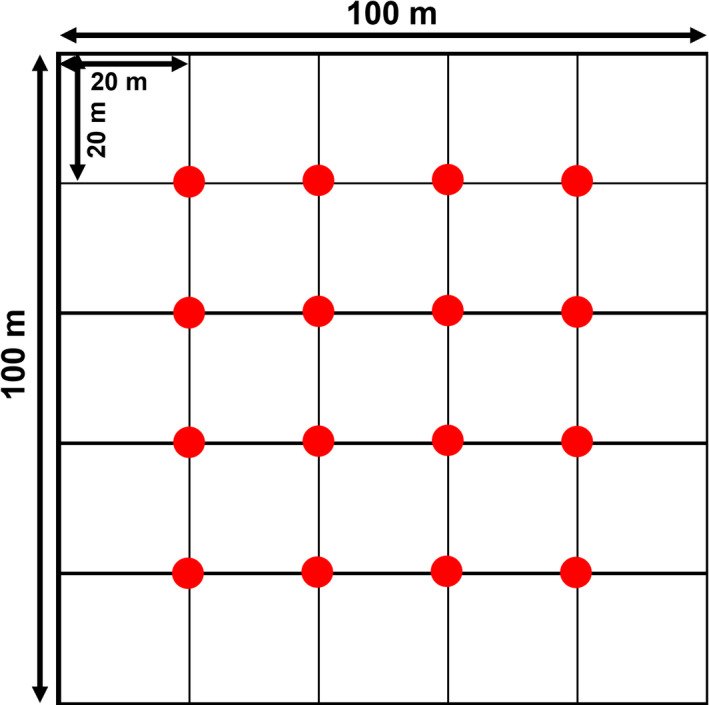
Diagram showing the grided study plot in each forest stage. Circles show the locations from which sampling points were randomly chosen for the measurements

We performed the study in two different periods of contrasting rainfall, which we will refer to as “wet” and “dry” periods in the results. In each period, we conducted the measurements twice, each separated by at least a month (Figure 1b, red frames). In each forest stage, we established a 1‐ha plot and divided it into 20‐m × 20‐m subplots, as shown in Figure A2. Then, we randomly selected six sampling points within the 1‐ha plot and measured all study variables concurrently at each point during 1000–1500 h on sunny days. For SR, we used a portable photosynthesis system (TARGAS‐1, PP Systems) connected to a soil respiration chamber (SRC‐2 Soil Respiration Chamber, PP Systems). In this process, the SR rate, measured in g CO_2_ m^−2^ h^−1^, was calculated by measuring the rate of increase in CO_2_ concentration in the chamber over a period, which was set to 60 s. Before taking measurements, we installed a soil collar with a cross‐sectional area of 78 cm^2^, on each selected sampling point at 5‐cm depth in the soil, leaving it for at least 1 h prior to SR measurement. Before putting the soil respiration chamber on the soil collar, we removed small living plants and coarse litter from the soil surface within the collar to avoid measuring their respiration (Peng et al., 2015; Zhou et al., 2007). Simultaneously, ST was measured using a probe (STP‐2 soil temperature probe, PP Systems) at 10 cm depth near the soil collar. Soil moisture was measured at 5 cm depth from the soil surface using a probe (SM150T, DeltaT Devices). For each sampling point, all measurements of SR, ST and SM were repeated three times and then averaged to represent each sampling point. In addition, the unit of SR was converted to µmolCO_2_ m^−2^ s^−1^ to facilitate the comparisons with other studies which mostly present the SR rate in this unit. For the soil analyses, we collected three 3.2‐cm diameter soil core samples from each study plot at 10‐cm soil depth in the wet season (September 2020) and the dry season (February 2021). We used a total organic carbon analyzer (Multi N/C 3100, Analytik Jena) to obtain OM values.

### 2.3 Statistical analysis

To answer the research questions, we analyzed differences in the measured variables across forest stages and between both periods. Before performing the data analysis, we used the Shapiro–Wilk test and Levene's test to check for normality and homogeneity of variance, respectively. For the comparison between two collection periods (wet and dry), we employed an independent *t* test for the data with normal distribution and the Mann–Whitney *U* test for nonnormal data. Then, for each period, we compared the SR, ST, SM, and OM across forest stages by using one‐way ANOVA with a Tukey's post hoc analysis for normally distributed data and the Kruskal–Wallis test with pairwise comparisons for nonparametric data. All statistical tests were performed in SPSS (IBM Corp. Released 2013. IBM SPSS Statistics for Windows, version 22.0). For the relationships among the variables, we performed regression analyses in SigmaPlot (version 12.0, Systat Software, Inc.) with SR as the dependent variable and ST, SM, and OM as the independent variables. In all statistical analyses, we used the significance level of 0.05.

## 3 RESULTS

**FIGURE 2:**
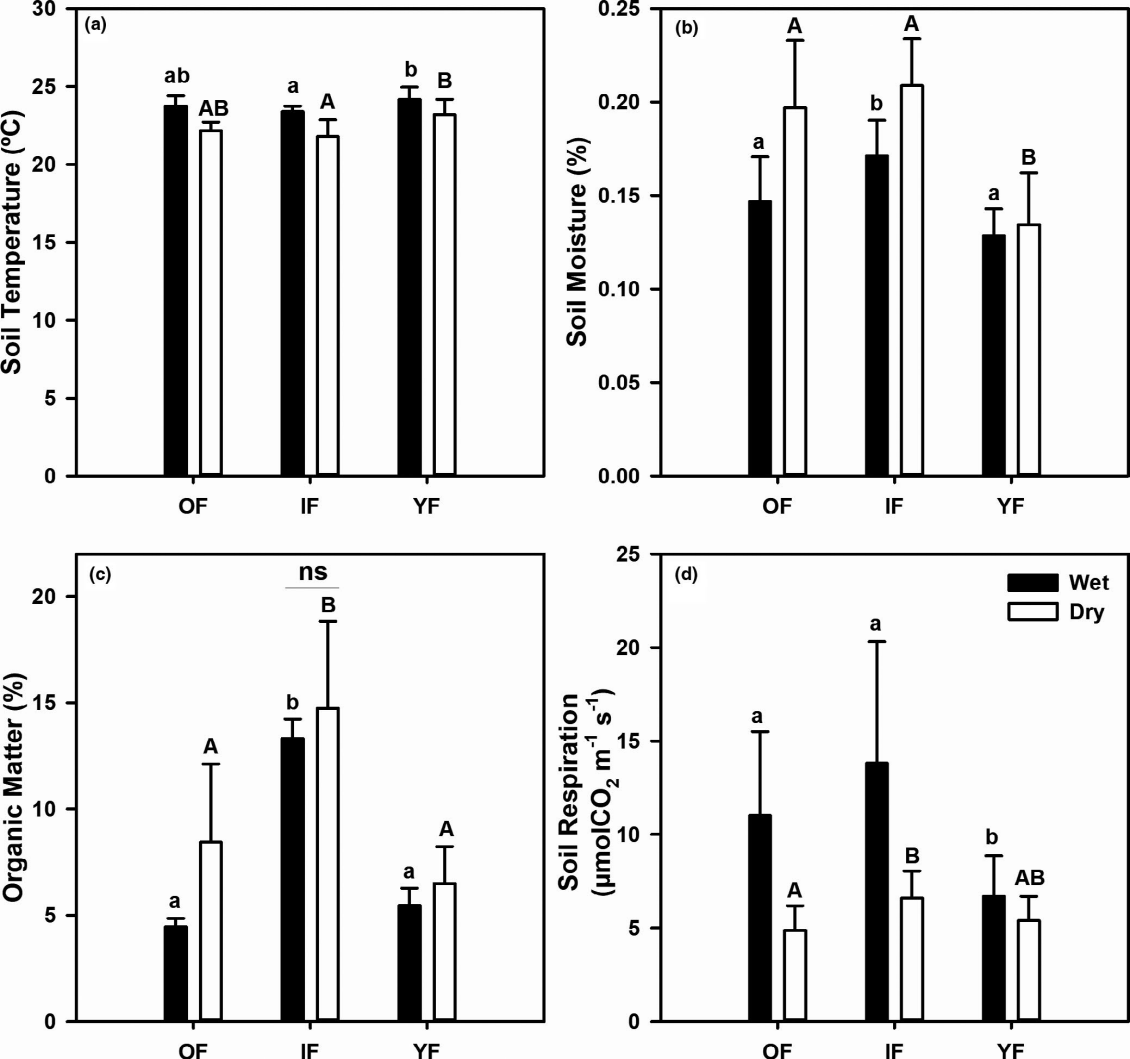
Mean values of the study variables including (a) soil temperature (°C), (b) soil moisture, (c) soil organic matter (%), and (d) soil respiration (µmolCO_2_ m^−2^ s^−1^) measured in the wet (filled bars) and the dry (open bars) periods in the old‐growth (OF), intermediate (IF) and young (YF) forest. Error bars indicate one standard deviation. Different small (capital) letters denote statistical differences among sites during the wet (dry) period at 5% significance level from the Tukey post hoc test or pairwise comparisons. All values significantly differed between periods, except the organic matter content in IF as indicated by “ns” or “not significant” in (c)

Figure 2 shows data of all measured variables, including soil temperature (ST), soil moisture (SM), soil organic matter (OM), and soil respiration (SR) during both collection periods. In both periods, ST in YF was significantly higher than in IF (Kruskal–Wallis, *H* = 8.074, *p* < .05 and *H* = 7.803, *p* < .05 for wet and dry periods, respectively), although it was not significantly different from that in OF. Soil temperature in all forest stages was significantly lower in the dry period in February and March 2021 (Mann–Whitney *U*, *U* = 164.000, *p* < .0001, Figure 2a), with an average of 22.4 ± 1.1°C (one standard deviation) than in the wet period in June and September 2020, with an average of 23.7 ± 0.7°C. Variations in SM across successional stages was observed across periods. During the dry period, SM in OF and IF was significantly higher than that in YF (one‐way ANOVA, *F* = 21.25, *p* < .0001), whereas in the wet period, SM in IF was the highest (one‐way ANOVA, *F* = 14.31, *p* < .0001). Overall, SM was significantly higher (independent *t* test, *t* = −3.656, *p* < .005, Figure 2b) in the dry period (average 0.18 ± 0.04) than that in the wet period (average 0.15 ± 0.03). The OM content was significantly higher in IF than in the other stages in the wet (Kruskal–Wallis, *H* = 28.125, *p* < .0001, Figure 2c) and the dry period (Kruskal‐Wallis, *H* = 17.843, *p* < .0001, Figure 2c). For each forest stage, the average OM content showed temporal variation in OF and YF, with higher values in the dry period (Mann–Whitney *U*, *U* = 132.000, *p* < .0001 and *U* = 108.00, *p* < .05 for OF and YF, respectively), whereas OM in IF was similar across periods (*p* = .843). Finally, in the wet period, SR in YF was significantly lower than that in other stages (Kruskal–Wallis, *H* = 10.572, *p* = .005). In the dry period, SR in YF did not differ from the older stages, but SR in OF was significantly lower than that in IF (one‐way ANOVA, *F* = 5.053, *p* = .012, Figure 2d). SR was significantly higher in the wet period than in the dry period in all stages (Mann–Whitney *U*, *U* = 245.000, *p* < .0001, Figure 2d). Overall, SR and its driving factors varied differently across forest stages and periods of data collection.

**FIGURE 3:**
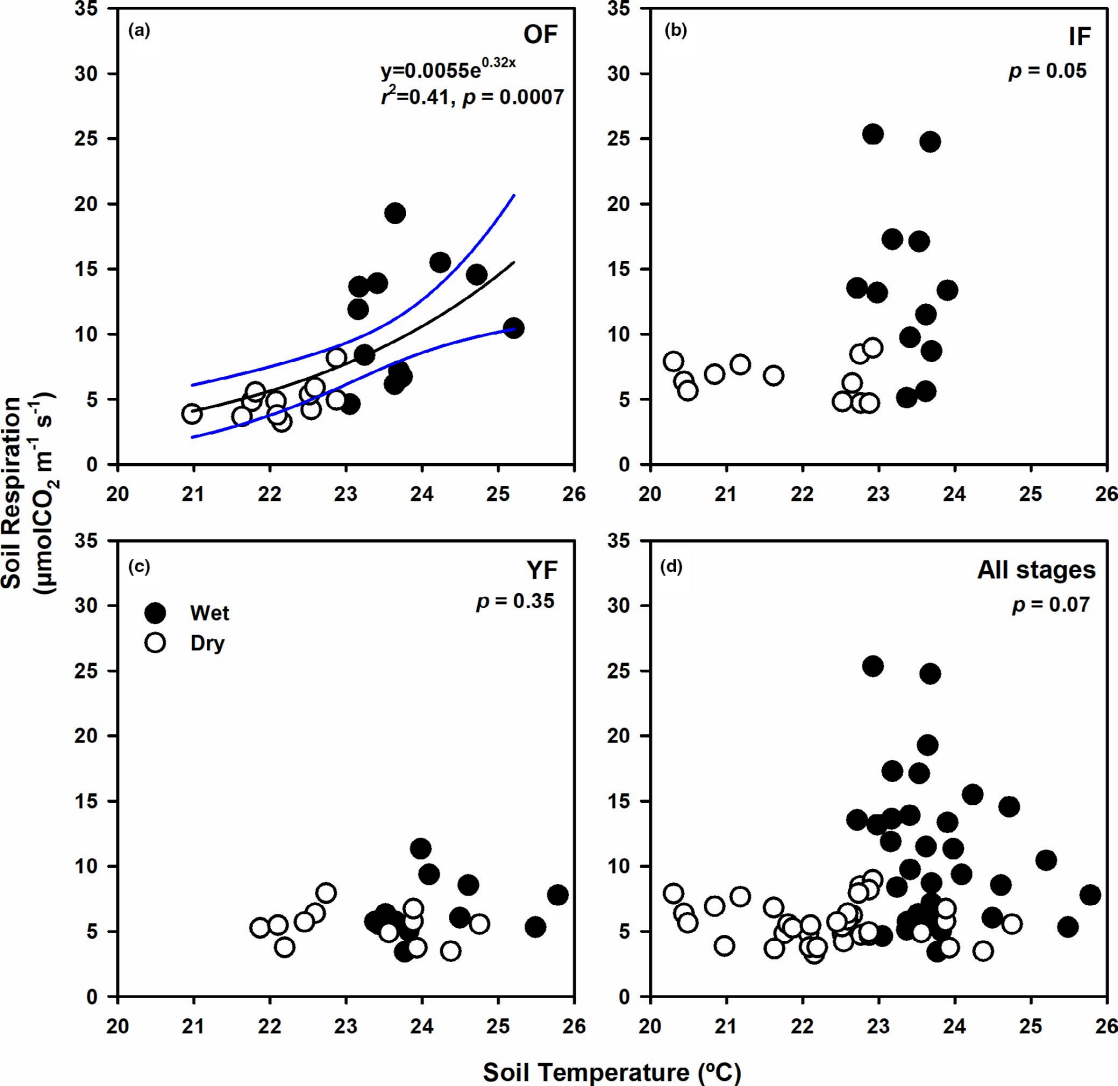
Relationships between soil respiration (µmolCO_2_ m^−2^ s^−1^) and soil temperature (°C) in the (a) old‐growth (OF), (b) intermediate (IF), (c) young (YF) forest, and (d) all forest stages. Closed (open) circles represent data from the wet (dry) period. Results from regression analysis for data combined across periods are shown accordingly. Black solid line indicates the significant regression result with 95% confidence intervals shown as blue lines. The significance level for the regression analysis was 0.05

Next, we analyzed the relationships between SR and its driving factors including ST, SM, and OM. Considering each successional stage with data from both periods, SR in OF exponentially increased with ST (*p* = .0007, Figure 3a), whereas SR in IF and YF did not respond to changes in ST (*p* ≥ .05, Figure 3b,c). Regardless of forest stages, SR did not respond to ST (*p* = .07, Figure 3d).

**FIGURE 4:**
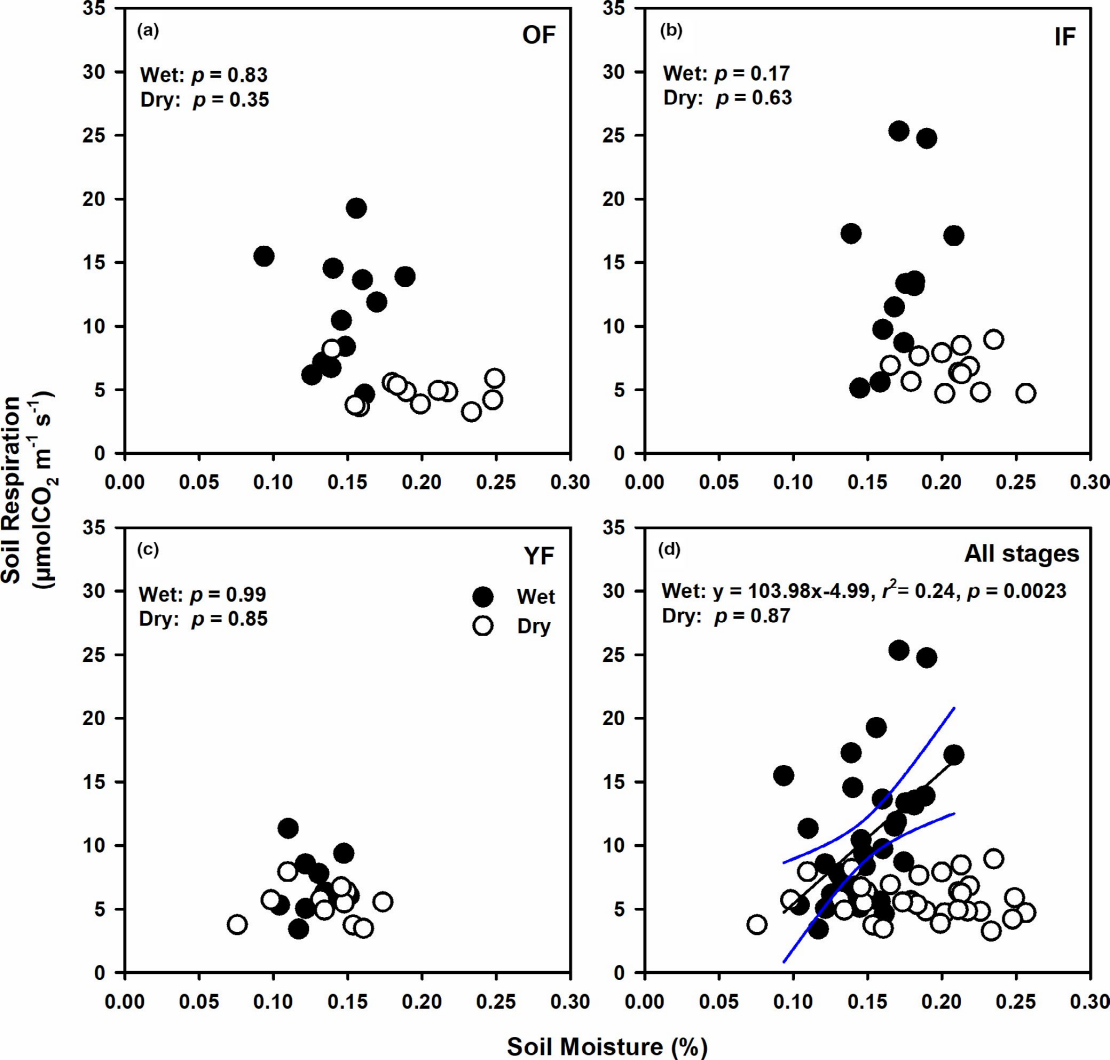
Relationships between soil respiration (µmolCO_2_ m^−2^ s^−1^) and soil moisture in the (a) old‐growth (OF), (b) intermediate (IF), (c) young (YF) forest, and (d) all forest stages. Closed (open) circles represent data from the wet (dry) period. Results from regression analysis for data combined across periods are shown accordingly. Black solid line indicates a significant regression result with 95% confidence intervals shown as blue lines. The significance level for the regression analysis was 0.05

Considering the relationships between SR and SM separately for each forest stage and period, no patterns were observed (*p* ≥ .17, Figure 4a–c). However, across forest stages, SR linearly increased with SM in the wet period (*p* = .0023), whereas no such response was observed in the dry period (*p* = .87, Figure 4d).

**FIGURE 5:**
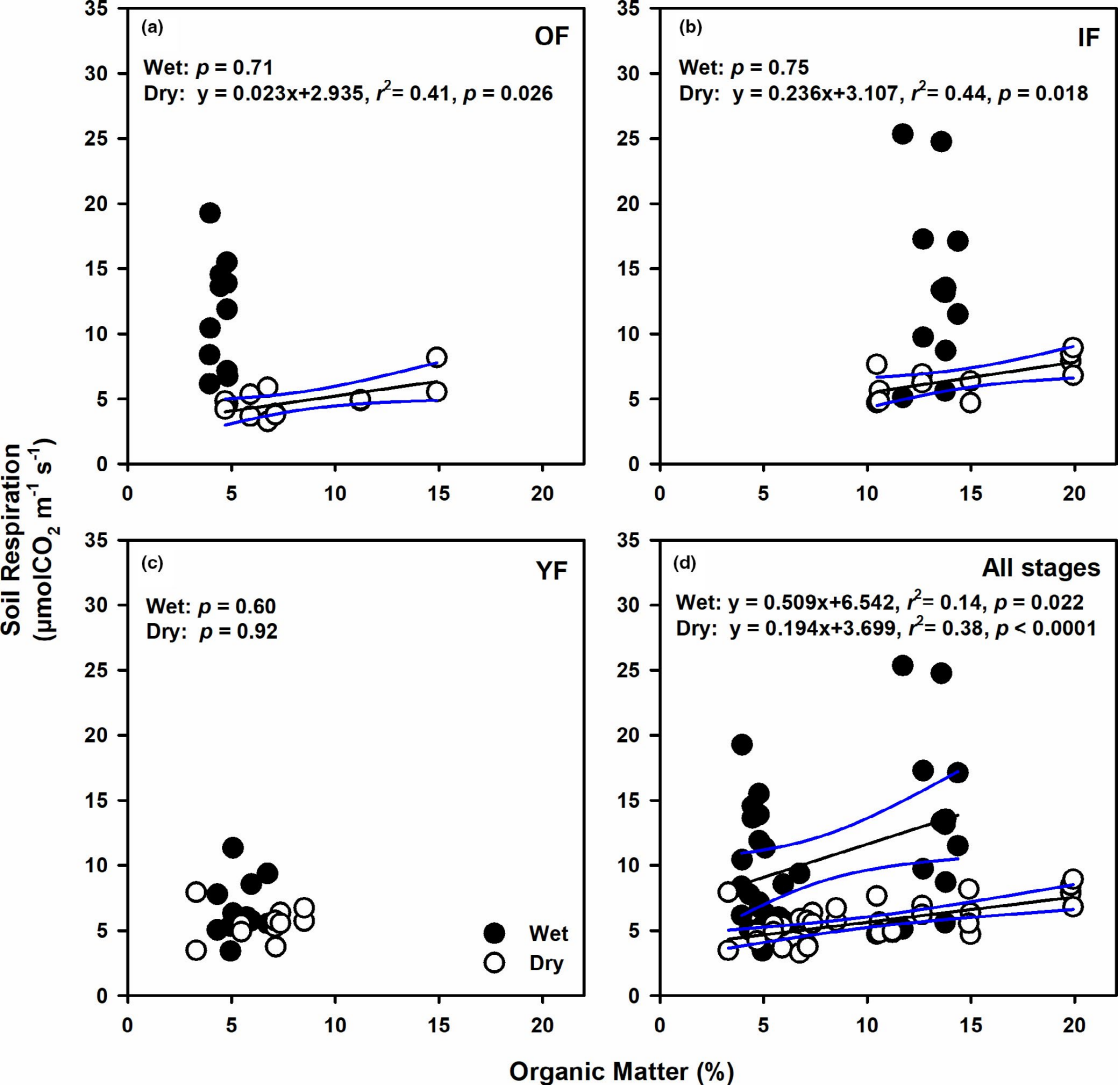
Relationships between soil respiration (µmolCO_2_ m^−2^ s^−1^) and soil organic matter (%) in the (a) old‐growth (OF), (b) intermediate (IF), (c) young (YF) forest, and (d) all forest stages. Closed (open) circles represent data from the wet (dry) period. Results from regression analysis for data combined across periods are shown accordingly. The black solid line indicates the significant regression result, with 95% confidence intervals shown as blue lines. The significance level for the regression analysis was 0.05

Across all forest succession and periods, SR linearly increased with OM, with stronger increasing rate in the wet period (*p* ≤ .022, Figure 5d). When analyzing the relationships separately by site, the response patterns were retained only in the dry period and in OF and IF (*p* ≤ .026, Figure 5a,b), whereas no responses were observed in YF (*p* ≥ .60, Figure 5c).

## 4 DISCUSSION

### 4.1 Comparison of SR from our study sites with reports from other forests in Southeast Asia

We summarized the SR values from previous studies in forests of Southeast Asia in Table A2. Our results could not be directly compared with any of these studies because it was unclear if any of these studies was conducted in similar seasonal evergreen forest. However, our SR values were within the ranges of those found in other Thai forests of various phenology, including dry evergreen forests (Adachi et al., 2009; Boonriam et al., 2021), dry dipterocarp forests (Hanpattanakit et al., 2015; Intanil et al., 2018), an evergreen forest (Hashimoto et al., 2004), a teak plantation (Kume et al., 2013), and a mixed deciduous forest (Takahashi et al., 2011). The SR values of our forests were also within the range of those from a lowland mixed dipterocarp forest in Malaysia (Katayama et al., 2009; Ohashi et al., 2008), whereas they were generally higher than those from forests at Pasoh, peninsular Malaysia (Adachi et al., 2005; Kosugi et al., 2007). Overall, it is evident that SR rates in Southeast Asian forests are highly variable and site‐specific.

### 4.2 Spatial variations in SR and the environmental factors across forest succession

Soil temperature showed spatial variation in both periods with higher values in the young forest than in the intermediate stage, although it was similar to that in the old‐growth forest. The higher ST in the young forest may be associated with its sparse canopy compared with the more closed canopy in the intermediate forest, as observed in our sites. The observations agreed with findings of higher ST in a Panamanian tropical forest with large forest gaps due to the direct heat from sunlight reaching the soil surface (Marthews et al., 2008). Our results showed that the differences in SM across forest stages varied temporally. In the dry period, soil moisture in YF was significantly lower than that in the older stages. However, in the wet period, IF had higher SM than that in the other sites. Again, canopy development may contribute to such variation because the canopy of YF was very sparse, whereas that of IF and OF was denser. Differing canopy density can affect the amount of light penetrating the soil surface and litterfall input to the soil, influencing surface evaporation and thus soil moisture. Overall, organic matter in the intermediate forest, with its high canopy density, was consistently higher in both periods than in that in the other sites. This may be explained by the high litterfall production in IF compared with the other two forests in our study (averaging 1.65, 2.08, 1.04 g m^−2^ day^−1^ in OF, IF, and YF, respectively, across wet and dry seasons; unpublished data), although other factors such as decomposition rate need to be considered to verify this claim. Although SR was generally similar in the older stages (OF and IF), it was significantly lowest in the young forest. This result agrees with previous studies indicating increasing soil respiration with forest age (Luo et al., 2012; Yan et al., 2006, 2009). Because soil carbon, which is highly correlated with soil organic matter, and soil moisture have been found to significantly explain variations in SR (La Scala et al., 2000; Stoyan et al., 2000), low materials for decomposition and consumption by the microbial community, and low soil moisture may contribute to the low SR in YF. Additionally, variation of root biomass may affect the difference in SR across forest stages, as related to total below‐ground carbon flux (TBCF; Katayama et al., 2009; Litton & Giardina, 2008). In fact, based on our preliminary measurements of fine root production in the older forests, we found that IF had higher fine root production than OF across both wet and dry periods (0.57 g m^−2^ day^−1^ in IF versus 0.50 g m^−2^ day^−1^ in OF), which was consistent with the higher SR in IF than in OF (Figure 2d).

### 4.3 Temporal variations in SR and the environmental factors between the wet and the dry period

Regardless of forest stage, ST was lower and SM was higher in the dry period than in the wet period, which may correspond to the cool dry season in this region. In addition, this may be attributed to low surface evaporation being blocked by the thick litter layer on the forest floor as observed through high monthly litterfall production in the dry season of the same study sites (averaged 2.07 and 1.10 g m^−2^ day^−1^ in the dry and the wet season, respectively, unpublished data). The low surface evaporation may be consistent with lower evapotranspiration during the (cool) dry season than in the wet season over the Chi and Mun river basins, where our site is located, as estimated from a process‐based model using satellite data from 2001 to 2015 (Zheng et al., 2019). Also, high litterfall production may have facilitated the retention of soil moisture because the increased volume of litter increased the time for soil drying or becoming saturated (Ogée & Brunet, 2002). Similarly, soil OM was generally higher in the dry period across forest succession, which may be associated with the higher litterfall in these sites during the dry season. In all forest stages, SR was significantly higher in the wet than in the dry period, which is consistent with previous studies on soil respiration in various forests in Thailand (Adachi et al., 2009; Boonriam et al., 2021; Hashimoto et al., 2004; Kume et al., 2013; Takahashi et al., 2011).

### 4.4 The influence of environmental factors on SR

To gain insights into the factors that play important roles in SR variation in these forests, we investigated the relationships between SR and the main drivers including ST, SM, and soil OM. Our results showed that ST and SM differently contributed to SR among forest stages and temporally, which was likely due to the inherent canopy and site characteristics of each stage. Overall, SR in our forests did not show a clear response to ST across both periods (Figure 3d). However, the general exponential relationship between SR and ST was significant only in the old‐growth, undisturbed forest (Lang et al., 2017). Because canopy gaps were unequally dispersed in OF, whereas those in IF and YF were more uniform, the range of ST was larger in OF across the wet and the dry period, possibly allowing high and significant variation of SR with ST (Figure 3a). In terms of soil moisture, SR of all forest stages increased with SM significantly only in the wet period (Figure 4d). This result indicated that low available soil moisture in the warm wet period constrained SR and thus was important for controlling microbial activity in these forests. In temperate and boreal forests, soil temperature has been identified as the major driver for soil respiration (Hursh et al., 2017). In fact, most models for soil CO_2_ efflux from these forests are empirical functions of soil temperature (Sugasti & Pinzón, 2020). In tropical regions, however, mixed results have been reported. Soil respiration of tropical forests is affected by both ST and SM in some sites (Boonriam et al., 2021; Ohashi et al., 2008; Sotta et al., 2006), only affected by ST in both primary and secondary sites of tropical montane forests in China (Zhou et al., 2013), and by only SM in various forests in Thailand (Adachi et al., 2005, 2009; Hashimoto et al., 2004; Kosugi et al., 2007; Takahashi et al., 2011). Another study has suggested that short‐term variation in SR depends on ST, but SM had greater effects on long‐term variation in SR in central Amazonian forests (Sotta et al., 2004). Therefore, the contribution of soil temperature and soil moisture to soil respiration rates in global forests varies greatly and is highly site‐specific with no clear spatial or temporal variation.

Our data showed significant increases in SR of most forest stages with increasing soil OM, with greater response in the dry period than in the wet period (Figure 5). Thus, the organic matter content in the soil was the main energy source for microbial activity that determined soil CO_2_ efflux in the dry period of these forests. As previously mentioned, this period corresponded to high litter addition to the forest floor, which may stimulate soil microbial activity as shown in greater soil CO_2_ release (Bréchet et al., 2017; Sayer et al., 2019, 2020). Large variations in OM were observed across forest stages, which may be explained by different quantity and quality of litter input (i.e., litterfall and roots) and different rates of litter decomposition in each stage. Note that the significant regression result for the wet period (Figure 5d) was mostly due to large differences in OM between IF and the other sites. Therefore, the observed significant pattern in the wet period may not represent the true response of SR to OM.

Overall, our results are still preliminary and suggest that different factors contribute to SR across spatial and temporal variation in our successional forests. In our forests, SM and OM were the limiting factors that significantly explained variation in SR in the wet and the dry period, respectively, whereas ST might explain variation in SR of the old‐growth forest with its nonuniform canopy compared with the younger forest stages. However, due to the limited data, further investigation including more sampling locations and higher frequency is needed to confirm these findings.

## 5 CONCLUSIONS

We investigated spatial and temporal variations in soil respiration (SR) and its driving factors including soil temperature (ST), soil moisture (SM), and organic matter content (OM), together with their relationships. Our analyses showed that SR was generally higher in the wet period and in older‐stage forests (either primary or secondary). Although ST has been identified as one of the main factors influencing SR in temperate and boreal forests, we found no significant relationships between SR and ST in our forests. However, in the old‐growth forest where gaps are usually nonuniformly scattered, ST and OM determined SR, and there were variations in response patterns across forest stages and periods. Across the successional forests, SM was the determining factor of SR in the wet period, whereas OM significantly explained SR variations in the dry period. Overall, the responses of SR to environmental factors were different across successional forests and data collection periods. Our results suggest the incorporation of different responses in successional forests and site‐specific information in modeling soil respiration of tropical forests. Nevertheless, detailed investigations involving long‐term and high‐frequency measurements and sampling locations should be performed to confirm these results.

## CONFLICT OF INTEREST

None declared.

## AUTHOR CONTRIBUTIONS

**Chadtip Rodtassana:** Conceptualization (equal); formal analysis (equal); funding acquisition (supporting); methodology (equal); writing–original draft (equal); writing–review and editing (equal). **Weerapong Unawong:** Methodology (supporting). **Siriphong Yaemphum:** Methodology (supporting). **Wirong Chanthorn:** Funding acquisition (supporting); methodology (supporting); project administration (equal); resources (equal); writing–review and editing (supporting). **Sakonvan Chawchai:** Funding acquisition (supporting); visualization (equal); writing–review and editing (supporting). **Anuttara Nathalang:** Funding acquisition (supporting); project administration (equal); resources (equal). **Warren Y. Brockelman:** Funding acquisition (supporting); project administration (equal); resources (equal); writing–review and editing (supporting). **Pantana Tor‐ngern:** Conceptualization (equal); formal analysis (equal); funding acquisition (lead); methodology (equal); writing–original draft (equal); writing–review and editing (equal).

## Data Availability

The raw data used in this study are publicly available in the Dryad repository at https://doi.org/10.5061/dryad.t4b8gtj2p

## APPENDIX A

**TABLE A1:** Soil particle distribution (mean ± SD) and soil texture classification of three forest successional stages at Khao Yai National Park, Thailand

Soil particle	Wet season (Sep 2020)			Dry season (Feb 2021)		
	OF	IF	YF	OF	IF	YF
Sand	53.7 ± 1.15^a^	37.7 ± 1.15^b^	64.4 ± 3.06^c^	40.3 ± 2.42^a^	35.7 ± 3.06^a^	56.4 ± 5.03^b^
Silt	16.9 ± 1.15^a^	26.3 ± 6.00^b^	12.3 ± 2.00^a^	27.7 ± 7.20^a^	30.3 ± 2.00^a^	13.6 ± 2.31^b^
Clay	29.3 ± 1.15^ab^	36.0 ± 5.03^a^	23.3 ± 1.15^b^	32.0 ± 7.57^a^	34.0 ± 1.15^a^	30.0 ± 3.06^a^
Soil texture	Sandy clay loam	Clay loam	Sandy clay loam	Clay loam	Clay loam	Sandy clay loam

Note

Different letters denote statistical difference among sites during each season at 5% significant level. The proportions of sand and silt in the soils of all sites significantly differed between seasons (*p* ≤ .017), but the percentage of clay in the soils did not vary between seasons (*p* = .219).

**TABLE A2:** Literature survey of soil respiration in Southeast Asian tropical forests including this study

Location	Vegetation type	Dominant tree species	MAT (°C)	MAP (mm)	Soil respiration (μmolCO_2_ m^−2^ s^−1^)	Reference
Pasoh area of Negeri Sembilan, Malaysia (2°5′N, 102°18′E, 75–150 m a.s.l.)	Primary and secondary mixed‐dipterocarp forest	Dipterocarp species	27	1,788	Primary forest: 2.78–6.93 Secondary forest: 2.58–6.36	Adachi et al. (2005)
Pasoh Forest Reserve, Malaysia (2°59′N, 102°18′E, 75–150 m a.s.l.)	Lowland mixed‐dipterocarp forest	*Shorea* and *Dipterocarpus* spp	25.4	1,800	2.5–6.5	Kosugi et al. (2007)
Lambir Hills National Park, Malaysia (4°20′N, 114°02′E, ~200 m a.s.l.)	Lowland mixed‐dipterocarp forest	Dipterocarp species	27	2,700	1.3–22.9	Ohashi et al. (2008); Katayama et al. (2009)
Huai Kha Khaeng, Thailand (15°20′N, 99°27′E, 549–638 m a.s.l.)	Dry evergreen forest	Dipterocarp species	23.5	1,242	Wet season: 3.15–9.99 Dry season: 1.24 –3.84	Adachi et al. (2009)
Sakaerat Environmental Research Station, Thailand (14°30′ N, 101°56′ E, ~500 m a.s.l.)	Dry evergreen forest	*Hopea ferrea* and *Hopea odorata*	26.7	1,751	Wet season: 4.3–13.3 Dry season: 1.8–6.8	Boonriam et al. (2021)
Ratchaburi Province, Thailand (35′13.3″N, 99°30′3.9″E, 110 m a.s.l.)	Dry dipterocarp forest	*Dipterocarpus intricatus*, *Dipterocarpus obtusifolius*, *Dipterocarpus tuberculatus*, *Shorea obtusa*, and *Shorea siamensis*	30.4	1,253	Wet season: 1.89–3.79 Dry season: 0.95–2.84	Hanpattanakit et al. (2015)
Kog‐Ma Experimental Watershed of Kasetsart University, Thailand (18°48′N, 98°54′E, ~1,300 m a.s.l.)	Evergreen forest	*Castanopsis*, *Lithocarpus*, and *Quercus* spp	20	2,084	2.13–14.07	Hashimoto et al. (2004)
Phayao Province, Thailand (19°02′14.38″N, 99°54′10.96″E, 512 m a.s.l.)	Dry dipterocarp forest	*Dipterocarpus obtusifolius*, *Dipterocarpus tuberculatus*, *Shorea obtusa*, and *Shorea siamensis*	25.1	936	0.77–1.13	Intanil et al. (2018)
Teak stand in Mae Moh plantation, Lampang Province, Thailand (18°25′N, 99°43′E, 380 m a.s.l.)	Teak plantations	Teak	25.8	1,284	0.96–5.02	Kume et al. (2013)
Mae Klong Watershed Research Station, Kanchanaburi province, Thailand (14°35′N, 98°52′E, 100–900 m a.s.l.)	Mixed deciduous forest	*Shorea siamensis*, *Vitex peduncularis*, *Dillenia parviflora* var. *Keruii*, and *Xylia xylocarpa* var. *Keruii*	25	1,650	Wet season; ridge: 4.00–8.25 upper slope: 5.17–6.89 lower slop: 4.20–9.97 Dry season; ridge: 1.33–4.42 upper slope: 2.18–5.67 lower slop: 2.50–8.09	Takahashi et al. (2011)
Khao Yai National Park, Thailand (14°26′N, 101°22′E, 700–800 m a.s.l.)	Primary and secondary seasonal evergreen forest	Old‐growth forest *Dipterocarpus gracilis,* *Sloanea sigun,* *Ilex chevalieri,* *Symplocos cochinchinensis,* *Schima wallichii* Intermediate forest *Schima wallichii,* *Machilus gamblei,* *Eurya acuminate,* *Symplocos cochinchinensis, Syzygium nervosum* Young forest *Cratoxylum cochinchinense, Syzygium antisepticum,* *Adinandra integerrima,* *Syzygium nervosum,* *Symplocos cochinchinensis*	22.4	2,100	Wet period; Old‐growth forest: 6.51–15.49 Intermediate forest: 7.28–20.32 Young forest: 4.50–8.86 Dry period; Old‐growth forest: 3.55–6.19 Intermediate forest: 5.13–8.05 Young forest: 4.09–6.69	This study

Note

Values are shown as ranges. MAT and MAP stand for mean annual temperature in °C and mean annual precipitation in mm, respectively.
